# *NPPB* and *ACAN*, Two Novel SHOX2 Transcription Targets Implicated in Skeletal Development

**DOI:** 10.1371/journal.pone.0083104

**Published:** 2014-01-08

**Authors:** Miriam Aza-Carmona, Veronica Barca-Tierno, Alfonso Hisado-Oliva, Alberta Belinchón, Darya Gorbenko-del Blanco, Jose Ignacio Rodriguez, Sara Benito-Sanz, Angel Campos-Barros, Karen E. Heath

**Affiliations:** 1 Institute of Medical and Molecular Genetics (INGEMM), Hospital Universitario La Paz, Universidad Autónoma de Madrid, IdiPAZ, Madrid, Spain; 2 Centro de Investigación Biomédica en Enfermedades Raras (CIBERER), Instituto Carlos III, Madrid, Spain; 3 Dept. Celular Biology, Immunology & Neurosciences, Facultad de Medicina, Universidad de Barcelona, Barcelona, Spain; 4 Department of Pathology, Hospital Universitario La Paz, Madrid, Spain; University of Massachusetts Medical, United States of America

## Abstract

SHOX and SHOX2 transcription factors are highly homologous, with even identical homeodomains. Genetic alterations in *SHOX* result in two skeletal dysplasias; Léri-Weill dyschondrosteosis (LWD) and Langer mesomelic dysplasia (LMD), while no human genetic disease has been linked to date with *SHOX2*. SHOX2 is, though, involved in skeletal development, as shown by different knockout mice models. Due to the high homology between SHOX and SHOX2, and their functional redundancy during heart development, we postulated that SHOX2 might have the same transcriptional targets and cofactors as SHOX in limb development. We selected two SHOX transcription targets regulated by different mechanisms: 1) the natriuretic peptide precursor B gene (*NPPB*) involved in the endochondral ossification signalling and directly activated by SHOX; and 2) Aggrecan (*ACAN*), a major component of cartilage extracellular matrix, regulated by the cooperation of SHOX with the SOX trio (SOX5, SOX6 and SOX9) via the protein interaction between SOX5/SOX6 and SHOX. Using the luciferase assay we have demonstrated that SHOX2, like SHOX, regulates *NPPB* directly whilst activates *ACAN* via its cooperation with the SOX trio. Subsequently, we have identified and characterized the protein domains implicated in the SHOX2 dimerization and also its protein interaction with SOX5/SOX6 and SHOX using the yeast-two hybrid and co-immunoprecipitation assays. Immunohistochemistry of human fetal growth plates from different time points demonstrated that SHOX2 is coexpressed with SHOX and the members of the SOX trio. Despite these findings, no mutation was identified in *SHOX2* in a cohort of 83 LWD patients with no known molecular defect, suggesting that *SHOX2* alterations do not cause LWD. In conclusion, our work has identified the first cofactors and two new transcription targets of SHOX2 in limb development, and we hypothesize a time- and tissue-specific functional redundancy between SHOX and SHOX2.

## Introduction

Alterations of *SHOX* and its enhancers have been reported in two skeletal dysplasias: Léri-Weill dyschondrosteosis (LWD, MIM 127300) and Langer mesomelic dysplasia (LMD, MIM 249700) as well as in a small proportion of individuals with idiopathic short stature (MIM 300582) [1]–[9]. Heterozygous mutations in *SHOX* or its enhancers results in LWD, a disproportionate short stature syndrome due to mesomelic shortening of the limbs, and the typical abnormality of the forearms known as Madelung deformity, characterized by the bowing of the radius and dorsal dislocation of the distal ulna. LMD is due to homozygous or compound heterozygous mutations in *SHOX* or its enhancers, resulting in severely disproportionate short stature with marked mesomelic and rhizomelic limb shortening. The estimated prevalence of SHOX haploinsufficiency is 1 in 1000 individuals [10].

The clinical symptoms produced by *SHOX* alterations, reflect its molecular function and also its expression pattern during limb development. SHOX belongs to the paired-related homeodomain family of transcription factors [11]. Two major SHOX isoforms exist, SHOXa and SHOXb [1], both containing a homeodomain (HD), but only SHOXa (which from now will be called in this paper SHOX) acts as a transcriptional activator in osteogenic cells via its transactivation domain, the OAR (Otp, Aristaless, Rax), which is absent in SHOXb. SHOX has been shown to regulate various genes involved in limb development: directly regulating the transcription of the natriuretic peptide precursor B gene (*NPPB*, MIM 600295) [12] which encodes the brain natriuretic protein (BNP), and fibroblast growth factor receptor 3 gene (*FGFR3*, MIM 134934) [13], two genes involved in the signalling of endochondral ossification. In contrast, SHOX cooperates with the SOX trio, via its protein interaction with SOX5/SOX6, to regulate the Aggrecan gene (*ACAN*, MIM 155760) which encodes a major component of cartilage extracellular matrix [14]. Recently, the homeobox transcription factor HOXA9, which is thought to be important in limb patterning [15], has been identified as the first regulator of *SHOX* expression [16].

During limb development, SHOX is principally expressed in the mesomelic portions of the limbs [17], [18], in the mesenchymal cells during the first steps of chondrogenesis [17], [18] and in the developing chondrocytes of the human growth plate throughout endochondral ossification [14], [19].

SHOX2 is the human paralog of *SHOX*, presenting a global homology of 83% and identical homeodomains [11]. Two SHOX2 isoforms exist, SHOX2a (which we call SHOX2 from now onwards) and SHOX2b, which differ only in their N-terminal regions [11]. However, the functional significance of these isoforms remains elusive.

Whilst no ortholog for *SHOX* exists in mice, an ortholog of *SHOX2* does exist [17]. The first *Shox2* knockout mouse was embryonically lethal, but it revealed the importance of this gene in palatogenesis and heart and limb development [20]. Subsequent analyses of different *Shox2* knockout mice and in xenopus showed that Shox2 participates in an intricate signalling pathway that regulates the sinoatrial node formation and pacemaking function [21]–[24].

Different conditional *Shox2* knockout mice models have showed severe rhizomelic limb but also mesomelic hindlimb shortening [20], [25], [26]. Shox2 regulates progression through chondrogenesis at two distinct stages, it prevents the onset of early chondrocyte differentiation and it controls the transition from mature to hypertrophic chondrocytes, regulating the expression of, among others, *Sox9*, *Sox6*, *Acan*, *Col2a1*, *Col10a1*, *Bmp2*, *Bmp4*, *Runx2* and *Runx3* [26], [27]. When *Shox2* is deleted from developing chondrocytes, Bmp4 expression is significantly increased, driving chondrocyte maturation and hypertrophy. However, when *Shox2* is deleted earlier in the limb bud, as in micromass cultures of *Shox2* mutant limb cells or in primary mouse bone marrow mesenchymal stem cells, only a modest increase in *Bmp4* expression occurs, sufficient to trigger early chondrogenesis but not enough to drive chondrocyte maturation and hypertrophy [26], [27].

We set out to determine whether SHOX2 could regulate the same transcription targets and interact both with SHOX and its cofactors, SOX5 and SOX6, during limb development as: 1) SHOX and SHOX2 are highly homologous; 2) both are expressed in the developing limbs, even overlapping in some regions [17], [18]; 3) they possess similar transcriptional activities [22]; and 4) functional redundancy between these proteins has been demonstrated in the regulation of sinoatrial node formation and pacemaking function [28].

Using luciferase reporter assays, we show that SHOX2, like SHOX, has the capacity to activate directly *NPPB* whilst requiring the SOX trio to activate *ACAN*. Moreover, using yeast two-hybrid assays and co-immunoprecipitation we have characterized the domains implicated in the dimerization of SHOX2 and its interaction with SHOX, SOX5 and SOX6. Immunohistochemistry of human fetal growth plates demonstrated that SHOX2 is coexpressed with SOX5, SOX6, SOX9 and SHOX. Taken together, our work has identified the first cofactors and two new transcription targets of SHOX2 in limb development.

## Materials and Methods

### Generation of recombinant constructs

The full-length cDNA of *SHOX2a* (NM_006884.3) was obtained by eliminating a 70 bp region of the IMAGE *SHOX2* clone 4122010 (Source Biosource, Berlin, Germany). The two regions of interest of this clone were amplified (PCRs A and B) and then fused by PCR ligation using appropriate oligonucleotides (Table S1 in File S1). The obtained product was subsequently cloned using the TA-cloning kit (Invitrogen, Life Technologies, Carlsbad, CA, USA) and then subcloned into different plasmids. The *SHOX2b* (NM_003030.4) and various *SHOX2a* fragments were amplified using the *SHOX2a* cloned region and appropriate oligonucleotides (Table S1 in File S1). The *SHOX2a* missense mutations and various *SHOX2a* fragments were created using the QuickChange Site-Directed Mutagenesis Kit (GE Healthcare, Fairfield, Co, USA), appropriate mutagenic oligonucleotides and different plasmids as templates (Table S2 in File S1). The SHOX, SOX5, SOX6 and SOX9 clones were as previously described [14].

The *NPPB* 1030 bp promoter was amplified using oligos and PCR conditions as previously described [12], cloned into the pCR2.1 vector (Invitrogen) and then subcloned into pGL3-Basic luciferase vector (Promega, Madison, WI, USA). The *Acan* enhancer plasmid (4XA1)pCol2Luc [29] was kindly donated by Dr Veronique Lefebvre.

### Cell culture

Human osteosarcoma (U2OS, ATCC HTB-96) and human embryonic kidney 293 cells (HEK293, ATCC CRL-1573) were maintained in Dulbecco's modified Eagle's medium (Invitrogen Gibco BRL), supplemented with 10% fetal bovine serum (Invitrogen Gibco BRL) and 1% penicillin and streptomycin (Invitrogen Gibco BRL). Cells were cultured at 37°C and 5% CO_2_.

### Luciferase assay

Luciferase assays were performed with U2OS cells as previously described [14]. Transient transfections in U2OS or stable U2OS cell lines expressing SHOX under an inducible system have proven to be very valuable tools allowing the initial characterization of the function of SHOX and SHOX2 and the identification of their target genes and cofactors [11]–[14], [16], [27], [30]. Basically, cells were transfected using FuGene (Roche Applied Bioscience, Switzerland) at a DNA:Fugene ratio of 2∶1 with different combinations of SHOX, SHOX2, SOX5/SOX6 and SOX9 expression plasmids and reporter vectors. In the case of *NPPB* reporter assays, 200 ng pRL-TK, 1500 ng reporter plasmid and 250 ng expression plasmids was employed whilst, in the *Acan* reporter assays, 1.5 ng pRL-SV40, 750 ng reporter vector and 125 ng expression plasmids were added. Samples were normalized, firstly, with respect to the Renilla luciferase activity and then to that transfected with the empty reporter plasmid. Each combination was transfected three times, and three biological replicates were analyzed. Statistical analyses were undertaken with SPSS v15.0 for Windows. We employed one-factor analysis of variance (ANOVA) with the Bonferroni post hoc test for analysing the results of luciferase assay.

### Yeast two-hybrid assay

The yeast two-hybrid assay was undertaken as previously described [14]. All values represent the mean and standard deviation of five independent transformation experiments, each performed in triplicate.

### Co-immunoprecipitation and Western blot analysis

Immunoprecipitations were carried out in HEK293 cells as previously described [14]. Western blot analyses were undertaken using the following rabbit polyclonal antibodies: anti-SHOX [30] at a dilution of 1∶3000, anti-SHOX2 (SAB2102137, Sigma Aldrich, St Louis, MO, USA) at a dilution of 1∶1000, anti-SOX5 (Ab26041, Abcam, Cambridge, UK) and SOX6 (Sc-20092, Santa Cruz Biotechnology, Dallas, TX, USA) at a dilution 1∶2000. No cross reactivity was observed between SHOX2 and SHOX (Fig. S1 in File S1).

### Immunohistochemistry

Human tibia growth plate sections were obtained from spontaneously aborted normal fetuses of 18, 27, 32 and 38 weeks, after obtaining ethical approval and informed consent. The detailed immunohistochemical procedure was as previously described [14]. Negative controls (Fig. S1 in File S1) were performed by: 1) replacing the primary antibody with PBS; 2) using a rabbit polyclonal IgG isotype control (Ab27472, Abcam) at a dilution of 1∶50; 3) incubating sections of normal adult colon (where SHOX2 protein is not expected to be expressed) with the SHOX2 antibody. Specificity of the employed SHOX, SOX5, SOX6 and SOX9 antibodies had been previously demonstrated [14].

### *SHOX2* mutation screening

Ethical approval was obtained from “Hospital Universitario La Paz”. All participants provided informed consent for the performed studies. The cohort consisted of 83 probands with LWD or suspected LWD. Clinical details were obtained for all patients recruited into the study. Whenever possible, these included birth details, anthropometric measurement, actual height and height standard deviation scores according to national standards [31], physical examination of extremities, and X-rays of the lower arm. Family histories were also documented, including parental heights. In all cases, the presence alterations in *SHOX* or its enhancers had been previously excluded [4], [7]–[9], [32], [33]. The control cohort consisted of 95 Spanish individuals with normal heights (Spanish DNA Bank, University of Salamanca).

Peripheral blood was drawn from probands for DNA extraction. Genomic DNA was isolated by the salt precipitation method [34]. The screening of point mutations, small deletions and insertions in the coding sequences and intron/exon boundaries of *SHOX2* (NM_006884.3) was performed using High Resolution Melting. DNA fragments were amplified using MegaMix-Gold (Microzone, Southampton, UK) and LC Green Plus + fluorescent dye (BioFire Diagnostics, Salt Lake City, UT, USA). PCR conditions are available in Table S3 in File S1. Melting curves of amplified samples were analyzed using LightScanner HR96 (BioFire Diagnostics). Subsequent sequencing of any sample with abnormal melting profiles was carried out using the BigDye Terminator V3.1 kit (Applied Biosystems, Foster City, CA, USA).

*SHOX2* deletions and duplications were studied using microsatellite markers or a self-designed MLPA (multiplex ligation-dependent probe amplification) assay. Microsatellite analysis was undertaken through the detection of heterozygosity at four different markers flanking *SHOX2* (Table S4 in File S1). PCR conditions were as previously described (4). The self-designed MLPA assay consisted of five *SHOX2* probes and three control fragments (Table S5 in File S1). MLPA reactions were carried out using EK1 SALSA MLPA Kit (MRC-Holland, Amsterdam, The Netherlands) according to the manufacturer's protocol. The ratios of the test's peak areas versus control's samples were determined subsequently. Normal peaks were classified as showing a ratio of 0.65–1.35 whilst deletions and duplications were classified as having a ratio <0.65 or >1.35, respectively.

## Results

### SHOX2 activates *NPPB* and *ACAN*

**Figure 1:**
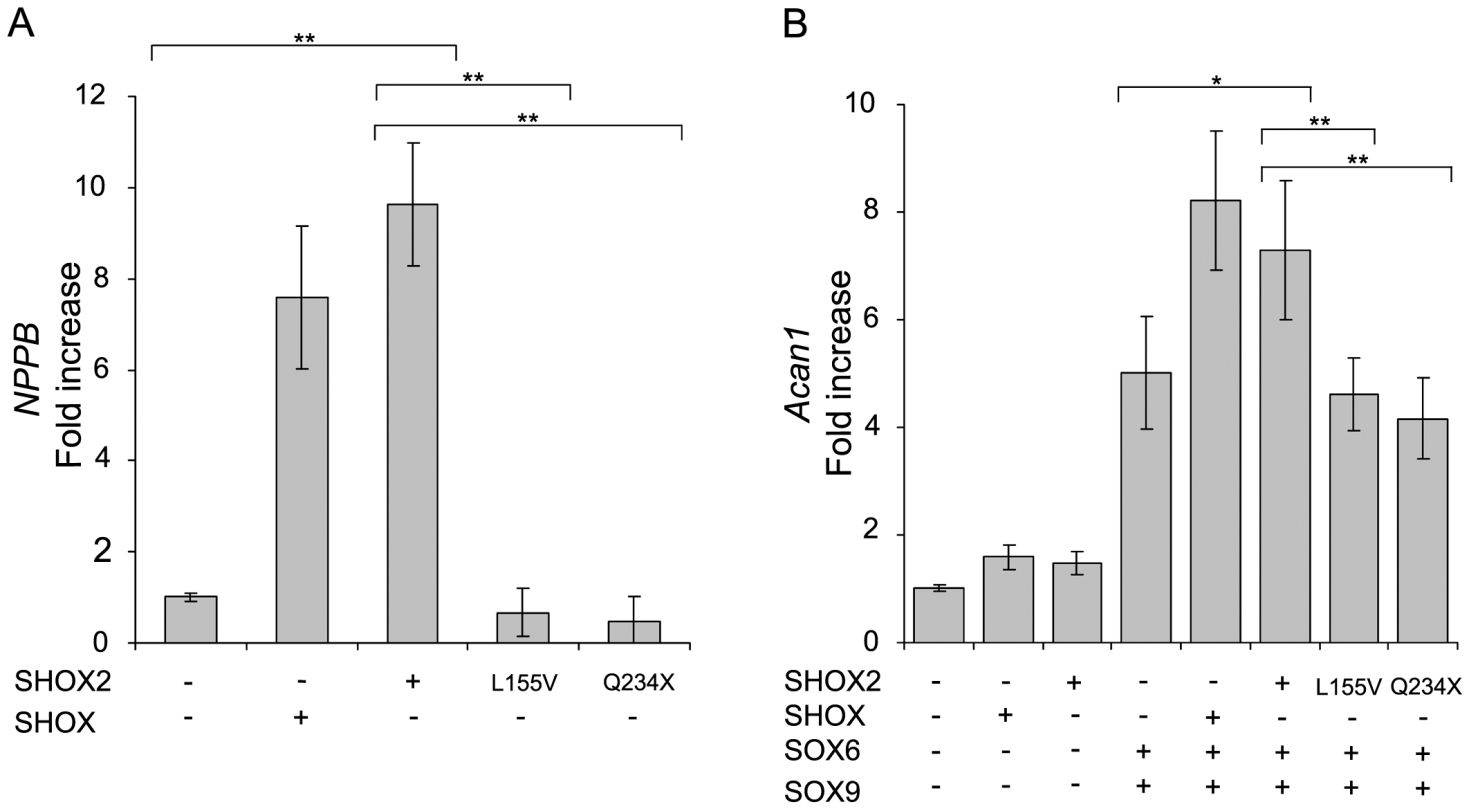
SHOX2 transactivates *NPPB* and *ACAN*. Luciferase reporter activity of U2OS cells transfected with reporter plasmids containing the *NPPB* promoter (A) or the *Acan* enhancer (B), renilla luciferase control plasmid and different combinations of SHOX, SHOX2, SHOX2(p.L155V), SHOX2(p.Q234X), SOX6 and SOX9 expression plasmid as indicated. Fold-increase values were obtained by normalizing the relative luciferase units of each sample with the relative luciferase units of the sample transfected only with the reporter plasmid. All values represent the mean and standard deviation of three independent samples, with each sample assayed in triplicate. Significant p-values obtained comparing different independent samples are indicated with an asterisk (*p<0.05 and **p<0.001).

We firstly analyzed the ability of SHOX2 to activate *NPPB*, a direct SHOX target (12), using a luciferase assay in U2OS cells. Cells overexpressing SHOX2 were cotransfected with a luciferase reporter plasmid carrying the *NPPB* promoter. SHOX2 was able to activate *NPPB* expression to an even greater degree than that observed for SHOX (Fig. 1A). With the purpose of confirming the SHOX2 activation, we included two SHOX2 mutants which mimic SHOX mutants reported in LWD patients (SHOX mutation database; http://hyg-serv-01.hyg.uni-heidelberg.de/lovd/index.php?select_db=SHOX): p.L155V that mimics the SHOX homeodomain p.L132V mutation, and p.Q234X, homolog to the SHOX p.Q211X mutation in which the OAR domain is absent. Both SHOX2 mutants failed to activate the *NPPB* promoter (Fig. 1A).

We subsequently set out to determine if SHOX2 was also able to activate the *Acan* enhancer via the SOX trio. Luciferase assays were undertaken using combinations of SHOX2, SOX5, SOX6 and SOX9. Due to the mutual redundancy of SOX5 and SOX6 [35] these proteins were included independently in the assays. As with SHOX, SHOX2 was able to activate *Acan* enhancer transactivation in cooperation with SOX6/SOX9 (Fig. 1B) and SOX5/SOX9 (Fig. S2 in File S1), but not directly (Fig. 1B and Fig. S2 in File S1). The two SHOX2 mutants, p.L155V and p.Q234X, reduced *Acan* enhancer activation (Fig. 1B and Fig. S2 in File S1), as observed with their SHOX homologues [14].

### SHOX2 isoforms dimerize

**Figure 2:**
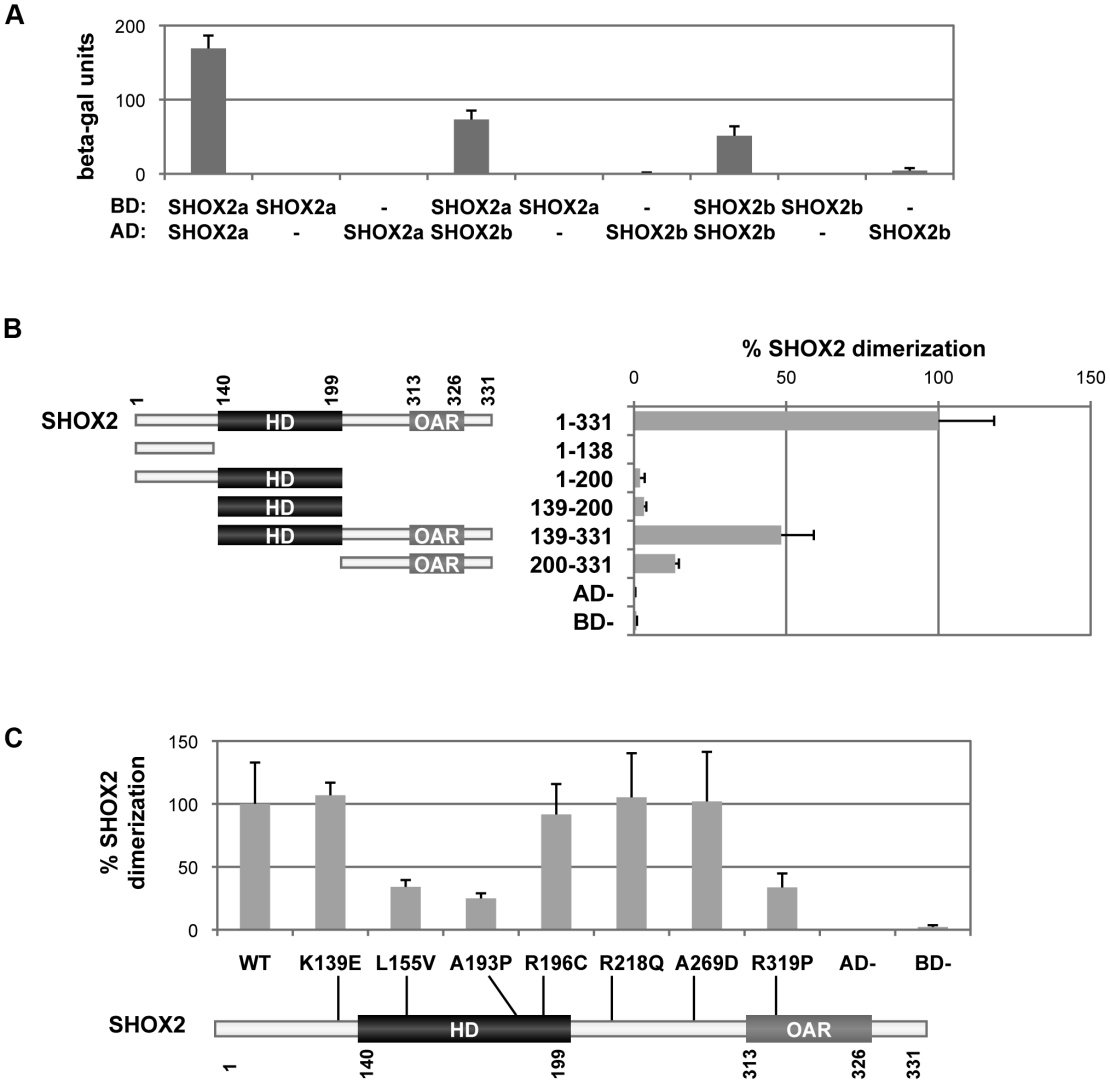
Identification and characterization of the SHOX2 dimerization. A) SHOX2a and SHOX2b homo- and hetero-dimerize in the yeast-two hybrid system. The S. cerevisiae strain Y187 was cotransformed with pGBT9 (containing the GAL4 binding domain – BD) and pACT2 (containing the GAL4 activation domain – AD) vectors. Interactions were determined using a β-galactosidase liquid assay with CRPG as substrate. Empty vectors were employed as negative controls. B) Characterization of the SHOX2 domains involved in the SHOX2 dimerization. The SHOX2 protein structure showing the amino acid location of the homeodomain (HD) and the OAR domain is shown with the various analysed SHOX2 fragments, indicating their name and amino acids that they contain. To the right of each fragment the corresponding yeast two-hybrid results are shown. Y187 cells were cotransformed with SHOX2 in the pGBT9 vector and SHOX2 fragments in the pACT2 vector. Protein interaction percentages were obtained by normalizing the β-galactosidase units of the different SHOX2 fragments to that obtained with full-length SHOX2. Empty vectors were employed as negative controls. C) SHOX2 mutants impair the SHOX2 dimerization. SHOX2 protein structure scheme showing the localization of the seven analysed missense mutations. Yeast two-hybrid assay of Y187 cotransformed cells with SHOX2 in the pGBT9 vector and the different SHOX2 mutants in the pACT2 vector. Protein interaction percentages were obtained by normalizing the β-galactosidase units of the various SHOX2 mutants to the wildtype SHOX2. Empty vectors were employed as negative controls.

Given that cooperative dimerization of paired-related homeodomains to DNA increases the transactivation efficiency to higher levels [36] and that SHOX preferentially binds to DNA as dimers [30], we assumed that SHOX2 should also dimerize to transactivate its target genes. Using the yeast two-hybrid assay, we verified that the SHOX2a and SHOX2b isoforms are capable of homo- and hetero-dimerization (Fig. 2A).

Using a series of SHOX2 deletion constructs (Fig. 2B) and the yeast two-hybrid assay, we observed that only the SHOX2(139–331) construct containing the homeodomain and the OAR domain clearly interacted with SHOX2 (Fig. 2B), whilst weaker interactions were observed for the constructs that contained only one of these two domains.

Subsequently, we analyzed the interaction capacity of wild-type SHOX2 with seven artificially designed SHOX2 missense mutations (Fig. 2C) that mimic SHOX mutations identified in LWD patients (SHOX mutation database, http://hyg-serv-01.hyg.uni-heidelberg.de/lovd/index.php?select_db=SHOX). Two homeodomain mutants (p.L155V and p.A193P) together with the OAR mutant p.R319P diminished their interaction capacity with SHOX2 (Fig. 2C). Interestingly, the SHOX2 p.R196C homeodomain mutant heterodimerizes with SHOX2 at similar levels to that of the wild-type (Fig. 2C), mimicking to that what was observed for its SHOX homologue, p.R173C [14], [37].

Thus, these experiments showed that both the homeodomain and the OAR are the domains implicated in the SHOX2 dimerization.

### SHOX2 interacts with SOX5 and SOX6

**Figure 3:**
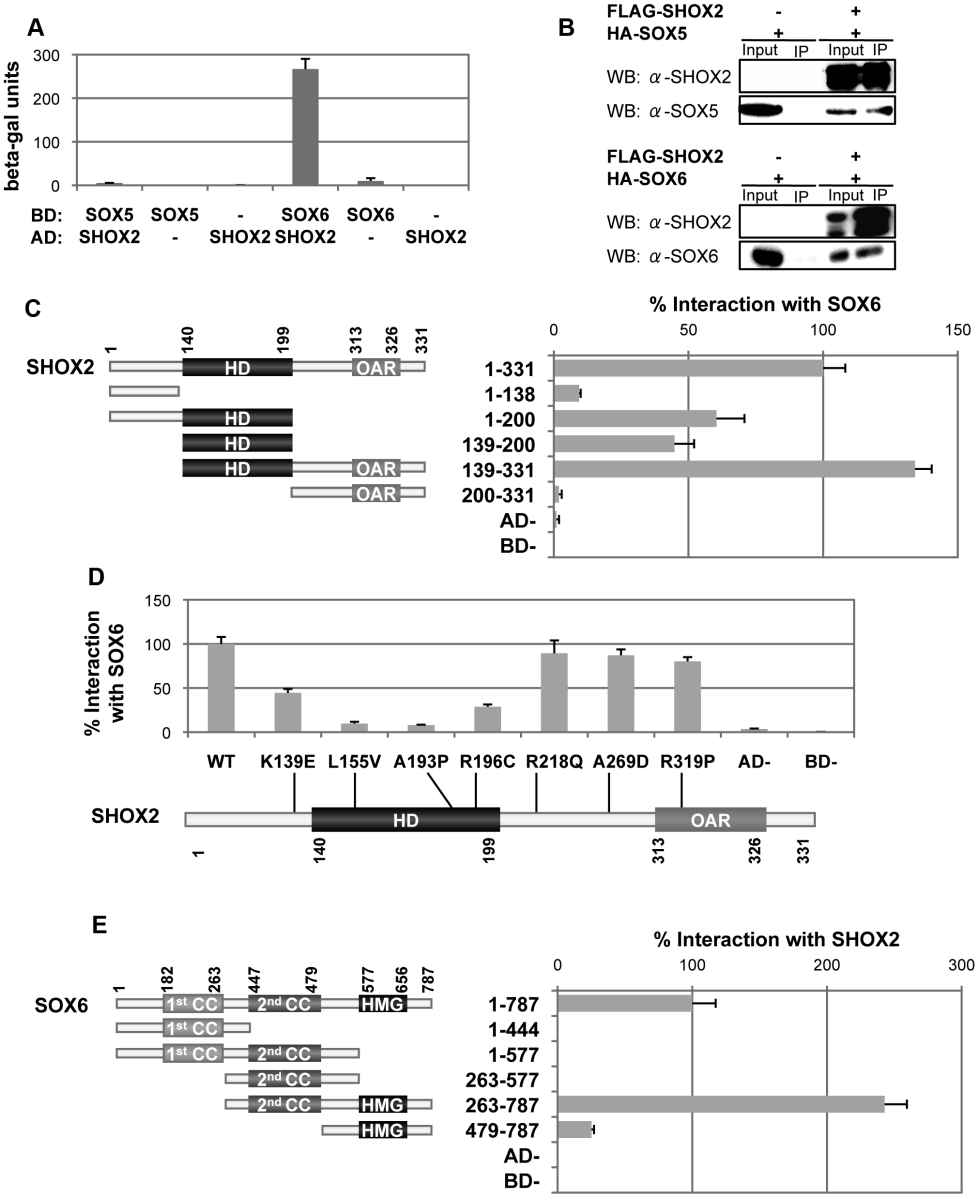
Identification and characterization of the SHOX2-SOX5 and SHOX2-SOX6 protein interactions. A) SHOX2 interacts with SOX5 and SOX6 in the yeast-two hybrid system. The S. cerevisiae strain Y187 was cotransformed with the pGBT9 (BD) and pACT2 (AD) vectors. Interactions were determined by using a β-galactosidase liquid assay with CRPG as substrate. Empty vectors were employed as negative controls. B) SHOX2 interacts with SOX5 and SOX6 in human cells. Nuclear extracts of HEK293 cells overexpressing FLAG:SHOX2 and HA:SOX5 or HA:SOX6 were immunoprecipitated using anti-FLAG-agarose. Western blots (WB) of immunoprecipitates (IP) were probed with SHOX2, SOX5 and SOX6 antibodies. Nuclear extracts corresponding to 10% input were included as protein expression controls and nuclear extract immunoprecipitates of cells transfected only with HA:SOX5 or HA:SOX6 were included as negative controls. The western-blot images clearly show that SOX5 and SOX6 immunoprecipitate only in the presence of SHOX2. C) Characterization of the SHOX2 domains involved in the interaction with SOX6. An scheme of the SHOX2 protein structure showing the amino acid location of the homeodomain (HD) and the OAR domain is shown with the various analysed SHOX2 fragments, indicating their name and the amino acids that they contain. To the right of each fragment, the corresponding yeast two-hybrid results are shown. Y187 cells were cotransformed with SOX6 in the pGBT9 vector and the SHOX2 fragments in the pACT2 vector. Protein interaction percentages were obtained by normalizing the β-galactosidase units of the different SHOX2 fragments to that obtained with full-length SHOX2. Empty vectors were employed as negative controls. D) SHOX2 mutants impair the interaction with SOX6. SHOX2 protein structure scheme showing the localization of the seven missense mutations analyzed. Yeast two-hybrid assay of Y187 cotransformed cells with SOX6 in the pGBT9 vector and the different SHOX2 mutants in the pACT2 vector. Protein interaction percentages were obtained by normalizing the β-galactosidase units of the various SHOX2 mutants to the wildtype SHOX2. Empty vectors were employed as negative controls. E) Characterization of SOX6 domains involved in the interaction with SHOX2 using the yeast two-hybrid assay. SOX6 is schematically drawn showing the amino acid location of two dimerization domains, the first and the second coiled-coils (1st cc and 2nd cc, respectively), and the HMG DNA-binding domain. Depicted below are the SOX6 generated constructs, indicating the name of each fragment and the amino acids that they contain. Y187 cotransformed cells with the different SOX6 fragments in the pGBT9 vector and SHOX2 in the pACT2 vector. The protein interaction percentages were obtained by normalizing the β-galactosidase units of the different SOX6 fragments to that obtained with full-length SOX6. Empty vectors were employed as negative controls.

Previously we had demonstrated that SHOX interacts with SOX5/SOX6 to activate the *Acan* enhancer in combination with SOX9 [14], thus, we proposed that as SHOX2 also increases *Acan* expression, it could interact with the SOX trio in a similar manner. The yeast two-hybrid assay showed that SHOX2 interacts strongly with SOX6 whilst very weakly with SOX5 (Fig. 3A). Co-immunoprecipitation in HEK293 cells clearly demonstrated that SHOX2 interacts with both SOX5 and SOX6 (Fig. 3B).

Given that SOX5 and SOX6 are highly homologous, with an overall homology of 67% and 90% identity in their HMG and coiled-coil domains [38], we decided to characterize only the SHOX2-SOX6 interaction using the yeast two-hybrid assay and a series of SHOX2 deletion constructs (Fig. 3C), SHOX2 missense mutations (Fig. 3D) and SOX6 deletion constructs (Fig. 3E). The three SHOX2 deletion constructs that were able to interact with SOX6, SHOX2(1–200), SHOX2(139–200) and SHOX2(139–331), share the homeodomain (Fig. 3C). The assay with various SHOX2 missense mutations confirmed that the homeodomain is involved in the SHOX2-SOX6 interaction since all the homeodomain mutants (p.L155V, p.A193P and p.R196C) diminished their interaction ability (Fig. 3D). Moreover, the p.K139E mutant, located in the amino acid adjacent to the homeodomain, also reduced its interaction capacity with SOX6 (Fig. 3D), suggesting that this amino acid is important for the interaction, as observed for its SHOX homologue p.K116E, which had a reduced interaction with SOX6 [14]. Although the SOX6(479–787) construct containing the HMG domain alone is able to interact with SHOX2 (Fig. 3E), the fact that the SOX6(263–787) construct containing both the HMG and the second coiled-coil domain interacts with SHOX2 at a greater strength than the SOX6 full protein, suggests that the second coiled-coil may confer structural stability to the HMG domain.

Therefore, the SHOX2 homeodomain and the SOX6 HMG domain are involved in the SHOX2-SOX6 interaction.

### SHOX2 interacts with SHOX

**Figure 4:**
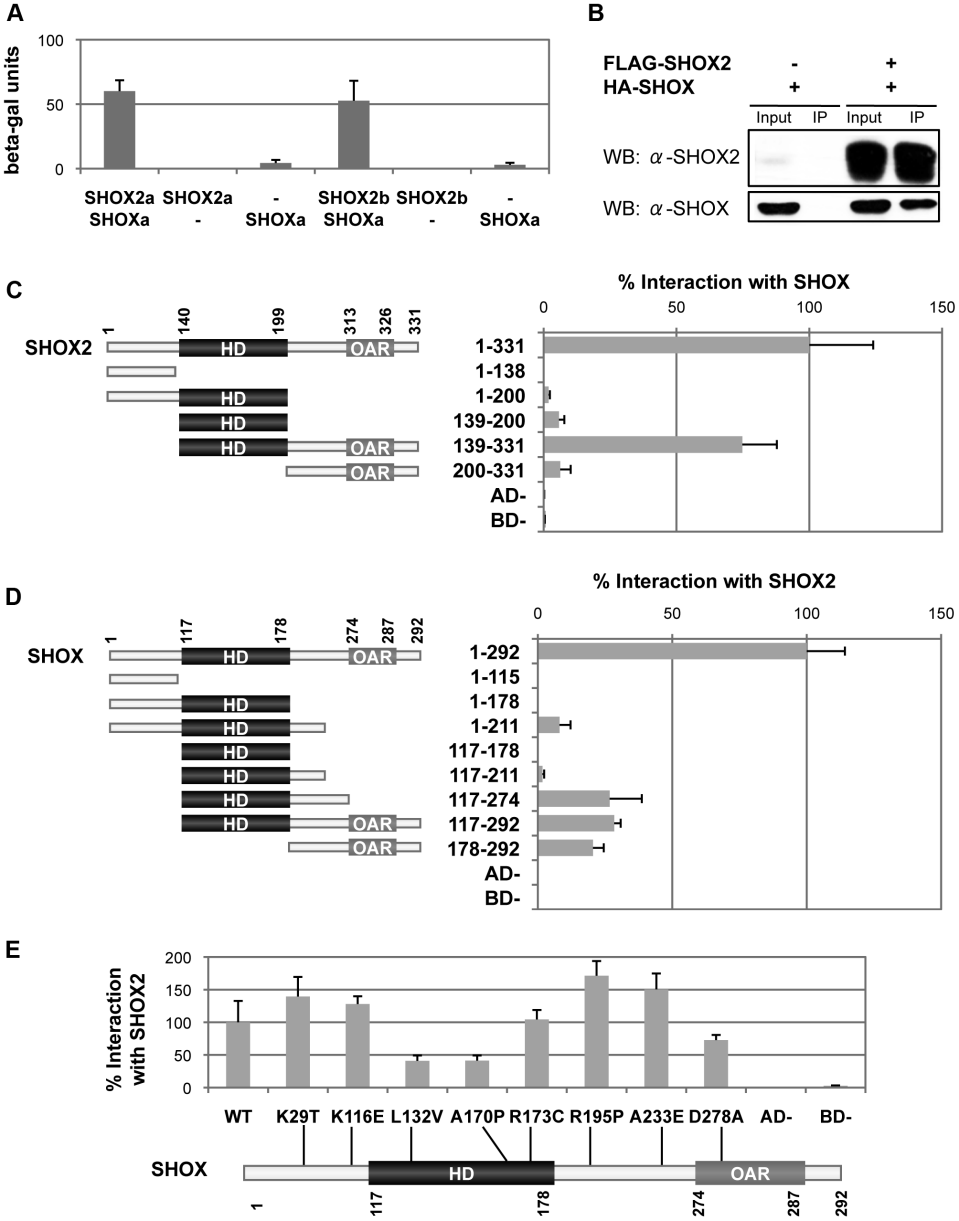
Identification and characterization of the SHOX2-SHOX interaction. A) SHOX2 interacts with SHOX in the yeast-two hybrid system. The S. cerevisiae strain Y187 was cotransformed with the pGBT9 (BD) and pACT2 (AD) vectors. Interactions were determined by using a β-galactosidase liquid assay with CRPG as substrate. Empty vectors were employed as negative controls. B) SHOX2 interacts with SHOX in human cells. Nuclear extracts of HEK293 cells overexpressing FLAG:SHOX2 and HA:SHOX were immunoprecipitated using anti-FLAG-agarose. Western blots (WB) of immunoprecipitates (IP) were probed with SHOX2 and SHOX antibodies. Nuclear extracts corresponding to 10% input were included as protein expression controls and nuclear extract immunoprecipitates of cells transfected only with HA:SHOX were included as negative controls. The western-blot images clearly show that SHOX immunoprecipitate only in the presence of SHOX2. C) Characterization of the SHOX2 domains involved in the interaction with SHOX. A scheme of the SHOX2 protein structure showing the amino acid location of the homeodomain (HD) and the OAR domain is drawn together with the various SHOX2 fragments analysed, indicating the name of each fragment the amino acids that they contain. Yeast two-hybrid assay of Y187 cells cotransformed with SHOX in the pGBT9 vector and different SHOX2 fragments in the pACT2 vector. Protein interaction percentages were obtained by normalizing the β-galactosidase units of the different SHOX2 fragments to that obtained with full-length SHOX2. Empty vectors were employed as negative controls. D) Characterization of the SHOX domains involved in the interaction with SHOX2 using the yeast two-hybrid assay. An scheme of the SHOX protein structure showing the amino acid location of the homeodomain (HD) and the OAR domain is shown with the various SHOX fragments analysed, indicating their name and the amino acids that they contain. To the right of each fragment are the corresponding yeast two-hybrid results. Y187 cells were cotransformed with the different SHOX fragments in the pGBT9 vector and with SHOX2 in the pACT2 vector. Protein interaction percentages were obtained by normalizing the β-galactosidase units of the different SHOX fragments to that obtained with full-length SHOX. Empty vectors were employed as negative controls. E) SHOX mutants impair the SHOX2-SHOX interaction. Schematic structure of SHOX showing the homeodomain (HD), the OAR domain and the localization of the eight analyzed missense mutations. Yeast two-hybrid assay of Y187 cotransformed cells with the different SHOX mutants in the pGBT9 vector and SHOX2 in the pACT2 vector. Protein interaction percentages were obtained by normalizing the β-galactosidase units of the various SHOX mutants to the wildtype SHOX. Empty vectors were employed as negative controls.

Due to the high homology and the overlapping expression pattern in limb development of SHOX and SHOX2, we set out to determine if they could interact between each other. Using the yeast two hybrid assay we identified that the two SHOX2 isoforms could interact with SHOX *in vivo* (Fig. 4A). We confirmed the SHOX2a-SHOX interaction by co-immunoprecipitation of HEK293 nuclear lysates overexpressing SHOX2a and SHOX (Fig. 4B).

Subsequently, we determined the domains implicated in this interaction employing deletion constructs (Fig. 4C–D) and SHOX missense mutations (Fig. 4E) in the yeast two hybrid assay. Only the SHOX2(139–331) construct containing the homeodomain and the C-terminal region including the OAR domain clearly interacted with SHOX (Fig. 4C), suggesting that both SHOX2 regions are required for the interaction with SHOX.

Among the different SHOX fragments examined, the SHOX(1–211), SHOX(117–274), SHOX(117–292) and SHOX(178–292) constructs were able to interact with SHOX2 (Fig. 4D) but to levels below 50% of the wildtype. The SHOX fragments appeared to be less stable and the SHOX interacting domain with SHOX2 was not clearly visible. Thus, further analysis was undertaken using eight SHOX missense mutations observed in LWD patients and located throughout the protein (Fig. 4E). The SHOX homeodomain mutants p.L132V and p.A170P showed a significant reduction in their interaction capacity with SHOX2 (Fig. 4E), whilst the OAR mutant p.D278A, had a reduced interaction but to a lesser extent (Fig. 4E). Therefore, the SHOX homeodomain appears to be the domain principally involved in the interaction with SHOX2 whilst the OAR domain appears to stabilize the interaction.

### SHOX2 is coexpressed with SHOX, SOX5, SOX6 and SOX9 in the human growth plate

Tissue and time dependent coexpression of proteins must occur for the interactions to occur between these proteins. Previous studies have shown that *SHOX2* expression overlaps with *SHOX* and *SOX9* in certain regions and stages of limb development [17], [18].

**Figure 5:**
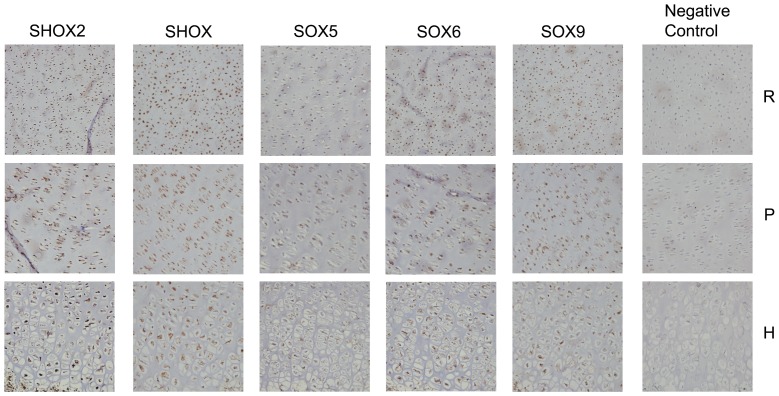
SHOX2 is coexpressed with SHOX, SOX5, SOX6 and SOX9 in the 18-week human fetal growth plate. Immunohistochemistry performed on 18-week human fetal tibia growth plates using antibodies against SHOX2, SHOX, SOX5, SOX6, SOX9 and the negative control (PBS). Specific staining can be observed in the reserve (R), proliferative (P) and hypertrophic (H) zones of the growth plate for all analysed proteins. Images performed at 20× magnification.

We firstly studied if SHOX2 is expressed in human fetal tibia growth plates of different developmental stages by immunohistochemisty, and showed that SHOX2 is expressed in the reserve, proliferative and hypertrophic zones at 18, 27, 32 and 38-weeks (Fig. S3 in File S1). Subsequently, we also demonstrated that SHOX2 is coexpressed with SHOX, SOX5, SOX6 and SOX9 proteins in the three differentiation regions of 18 and 38-weeks human fetal tibia growth plates (Fig. 5 and Fig. S4 in File S1).

### *SHOX2* mutations are not responsible for the LWD phenotype in our cohort of patients with unknown molecular defect

Defects in SHOX or its regulatory elements have been identified in ∼70% of LWD patients [33], [39], [40], whilst the remaining ∼30% remain genetically uncharacterized. Screening of the coding regions and intron-exon boundaries of *SHOX2* in 83 LWD patients with no known defect was performed using HRM. Samples with abnormal melting profiles were subsequently sequenced. Complete or intragenic *SHOX2* deletions/duplications were analysed using four microsatellite markers surrounding *SHOX2* (Table S4 in File S1) or by a self-designed MLPA assay (Table S5 in File S1).

Only two variants were identified, both located in exon 1 of *SHOX2*: a duplication of three glycines in the glycine-rich repeat, p.Gly77-Gly78dup (c.232_233dupGAGGAGGTG) and a missense mutation p.E21K (c.61G>A). We therefore evaluated the pathogenicity of these variants by determining their frequencies in control populations and by analyzing their cosegregation with the LWD phenotype when possible. In contrast to SHOX, where insertions and deletions of the homologous SHOX glycine repeat have been described in patients with LWD/ISS (SHOX mutation database, http://hyg-serv-01.hyg.uni-heidelberg.de/lovd/index.php?select_db=SHOX), the SHOX2 glycine repeat length appears to be a non-pathogenic CNV, since 183/7815 controls (Exome Variant Server) and 3/95 Spanish control individuals with normal heights carried this three amino acid insertion. The second mutation, p.E21K was assessed in the control population and in family members. Although the p.E21K mutation was only present in 2/12092 individuals (Exome Variant Server) and was absent in the 95 Spanish normal height controls, it did not cosegregate with the phenotype in the family (data not shown). Thus, *SHOX2* is not the molecular cause of the studied LWD cases.

## Discussion

We have demonstrated that SHOX2 regulates *NPPB* directly whilst activating *ACAN* via its cooperation with the SOX trio. The SHOX2 activation of the *NPPB* promoter was even higher than that observed for SHOX. Both analysed SHOX2 mutants, p.L155V with reduced dimerization capacity and the p.Q234X lacking the OAR domain, failed to upregulate *NPPB*, thus suggesting that the SHOX2 homeodomain and the transactivation domain are both critical for the *NPPB* activation.

A role for *NPPB* during endochondral ossification and longitudinal growth was initially suggested by the observation of skeletal overgrowth and alterations in the growth plate of *Nppb* transgenic mice [41]. Despite this, no further implication in skeletal development has been reported to date. With our new data, it would be interesting to test if *Nppb* expression is reduced in embryonic limb bud micromass cultures from *Shox2* mutant mice models. In contrast, the involvement of BNP in cardiovascular function is much clearer, to the extent that this peptide is a biomarker for diagnosis and prognosis of heart failure [42]. Given that *Shox2* participates in heart development as shown by different knockout mice models [20], [22], [23], it is tempting to speculate that *Shox2* also regulates *Nppb* expression in heart and that the disruption of this pathway is critical for the heart failure in *Shox2* knockout mice models.

The other SHOX2 transcriptional target discovered in this work is *ACAN*, which codifies for Aggrecan, a main component of the cartilage extracellular matrix. Previously, our group demonstrated that SHOX interacts with SOX5/SOX6 to cooperate with SOX9 in the activation of *ACAN* [19]. As with SHOX, SHOX2 cannot directly activate the *Acan* enhancer but requires the cooperation of the SOX trio. These results correlate with those observed in *Shox2* mutant mice where *Acan* levels were significantly lower in *Col2a1-CreShox2* mutant limb bud micromass cultures [26]. Further characterization demonstrated that the SHOX2 homeodomain and OAR domains are both critical for this *Acan* induction, since mutants in both domains failed to upregulate *Acan*. In this work, we have also demonstrated that SHOX2 interacts with SOX5 and SOX6. The SHOX2 homeodomain was shown to interact with the SOX6 HMG domain, thus in agreement with the results observed with SHOX-SOX6 [14] and other protein interactions [43]–[45]. Interestingly, the p.K139E mutant, located in the amino acid adjacent to the homeodomain of SHOX2, reduced its interaction capacity with SOX6 as observed previously with the homologous SHOX mutant, p.K116E [14], thus, implicating that this amino acid is also important for the interaction.

Our finding the homeodomain of SHOX and SHOX2 is implicated in the heterodimerization agrees with previous studies of other homeodomain proteins [46]. This interaction could: 1) alter the transactivation of their transcription targets by interacting with different cofactors and/or binding to DNA with different affinities; and 2) regulate different transcription targets, compared to that of the homodimers. More research is required in order to decipher the biological function of the discovered SHOX-SHOX2 protein interaction. In the future, it will be important to confirm these interactions and enlighten our knowledge further using *in vivo* models, such as the *SHOX/Shox2* knock-in mouse model [28], a skeletal-specific conditional knock-in mouse or chicken limb buds.

For these interactions to have physiological importance, SHOX and SHOX2 have to be coexpressed in a tissue and time dependent manner. It has been reported that SHOX is mainly expressed in the mesomelic portion of the limbs whilst SHOX2 expression occurs in the rhizomelic region, but overlapping expression has been observed [17], [18]. Further evidence of SHOX expression in the rhizomelic region has been shown in LMD individuals who lack SHOX, but present with both mesomelic and rhizomelic shortening of the limbs [47]. Shox2 expression has also been detected in the mesomelic parts of the limbs in mouse [25], [48], although it is difficult to extrapolate this data to humans due to the lack of a SHOX ortholog in mouse. In this work, we have demonstrated that SHOX and SHOX2, and also the SOX trio members are coexpressed in human tibial growth plates at different developmental stages. This data thus, argues for the coexpression of SHOX and SHOX2 proteins in some limb regions during specific time points.

Therefore, due to the high homology between SHOX and SHOX2 and their identical homeodomains, their overlapping expression in some limb regions, the sharing of transcriptional targets implicated in limb development (i.e. *NPPB* and *ACAN*) and the total rescue of the heart defects and its tissue-specific rescue in limbs when *SHOX* was expressed in the *Shox2*KI/KI mice [28], we argue for the existence of functional redundancy between SHOX and SHOX2 in a tissue-specific manner during human embryonic development. An interesting argument for this redundancy is shown by the LWD phenotype. In these patients, the skeletal deformities, i.e. the characteristic Madelung deformity, is more pronounced in the distal region of the upper limb, where it has been suggested that there is no SHOX-SHOX2 coexpression [17], and thus SHOX2 cannot rescue the SHOX haploinsuficiency. However, in the more proximal region of the radius and ulna, SHOX and SHOX2 appear to be coexpressed [17], thus, SHOX2 may be able to rescue the phenotype. But, in the case of LMD, SHOX2 is unable to rescue the phenotype caused by the complete absence of SHOX, thus, this rescue mechanism may have a limiting threshold. Further studies will be required to support this hypothesis.

As we have shown that SHOX2 shares cofactors and transcription factors implicated in skeletal growth, we screened for *SHOX2* alterations in a cohort of 83 patients with LWD or possible LWD with no known PAR1 defect. No pathogenic alteration was detected. Analyses of the Decipher database (http://decipher.sanger.ac.uk/) entries revealed the presence of eight patients with copy number variants (CNVs) of *SHOX2*. The majority of these patients had multiple CNVs located throughout the genome and no common clinical phenotype was observed. Only two patients presented with one large CNV at the *SHOX2* locus, but again no common phenotype was described. As SHOX2 appears to have a broad expression pattern [17] and the *Shox2*−/− mouse has heart, palate and skeletal defects [20], we postulate that individuals with *SHOX2* defects may present with a more severe phenotype: limb shortening, heart anomalies and/or cleft palate.

In summary, we have demonstrated that SHOX2, like SHOX, activates *NPPB* directly whilst activation of *ACAN* is through the cooperation with the SOX trio, adding further support to the theory that there is functional redundancy between SHOX and SHOX2 during human embryonic development in a tissue-specific manner.

## Supporting Information

File S1 **File includes Figures S1–S4 and Tables S1–S5.** Fig. S1: Specificity of the different antibodies employed. A) Immunohistochemical controls performed in 38-wk fetal growth plates and adult normal colon sections: PBS - primary antibody replaced by PBS, Isotype - rabbit polyclonal IgG isotype control antibody, SHOX2 – SHOX2 antibody incubated with sections from adult normal colon where this protein is not expected to be expressed. Note the negative staining for the majority of the cells. Images performed at 20× magnification. B) Immunoblots showing the specificity of the SHOX2 antibody. Nuclear extracts of HEK293 cells overexpressing SHOX, SHOX2, SOX5, SOX6 and SOX9 were separated on SDS polyacrylamide gels and probed with anti-SHOX2. Anti-GAPDH was used as loading control. Fig. S2: SHOX2 cooperates with SOX5 and SOX9 to activate the *Acan* enhancer. Luciferase reporter activity of U2OS cells transfected with a reporter plasmid containing the *Acan* enhancer, renilla luciferase control plasmid and different combinations of SHOX, SHOX2 WT, SHOX2(p.L155V), SHOX2(p.Q234X), SOX5 and SOX9 expression plasmid as indicated. Fold-increase values were obtained by normalizing the relative luciferase units of each sample with the relative luciferase units of the sample transfected only with the reporter plasmid. All values represent the mean and standard deviation of three independent samples, with each sample assayed in triplicate. Significant p-values <0.001 obtained comparing different independent samples are indicated with two asterisks. Fig. S3: Expression of SHOX2 in different stages of the human fetal growth plate. Inmunohistochemistry performed with anti-SHOX2 antibody in normal fetal growth plates of 18, 27, 32 and 38-weeks. SHOX2 is expressed in the reserve (R), proliferative (P) and hypertrophic (H) chondrocytes. Images performed at 10× magnification. Fig. S4: SHOX2 is coexpressed with SHOX, SOX5, SOX6 and SOX9 in the 38-week human fetal growth plate. Immunohistochemistry performed on 38-weeks human fetal tibia growth plates using antibodies against SHOX, SHOX2, SOX5, SOX6 and SOX9. Specific staining can be observed in the reserve (R), proliferative (P) and hypertrophic (H) zones of the growth plate for all the analysed proteins. Images performed at 20× magnification. Table S1: Oligonucleotide sequences for the amplification of the SHOX2a cDNA and the cloning of the SHOX2 constructs. The incorporated enzymes sites are indicated in small letters. SHOX2a PCR A antisense oligonucleotide and SHOX2a PCR B sense oligonucleotide flank the sequence to be eliminated. Table S2: Oligonucleotide sequences for the generation of various SHOX2 fragments and SHOX2 missense mutants. The mutated site is indicated by a small letter. Table S3: Oligonucleotide sequences for the mutation screening of the coding exons and intron/exon boundaries of *SHOX2*. Table S4: Oligonucleotide sequences, PCR conditions and amplicon sizes of the *SHOX2* microsatellite markers. Microsatellites are listed in order from telomere to centromere. Table S5: *SHOX2* self-designed MLPA. Chromosomal and *SHOX2* location, probe lengths and ligation site sequences are indicated for the *SHOX2* and three control fragments.
(PDF)
