# Supplementary material for: NPPB and ACAN, Two Novel SHOX2 Transcription Targets Implicated in Skeletal Development
Source: PLoS One. 2014 Jan 8;9(1):e83104. doi: 10.1371/journal.pone.0083104 (PMC3885427; doi:10.1371/journal.pone.0083104)
Supplement: File S1 — File includes Figures S1–S4 and Tables S1–S5. Fig. S1: Specificity of the different antibodies employed. A) Immunohistochemical controls performed in 38-wk fetal growth plates and adult normal colon sections: PBS - primary antibody replaced by PBS, Isotype - rabbit polyclonal IgG isotype control antibody, SHOX2 – SHOX2 antibody incubated with sections from adult normal colon where this protein is not expected to be expressed. Note the negative staining for the majority of the cells. Images performed at 20× magnification. B) Immunoblots showing the specificity of the SHOX2 antibody. Nuclear extracts of HEK293 cells overexpressing SHOX, SHOX2, SOX5, SOX6 and SOX9 were separated on SDS polyacrylamide gels and probed with anti-SHOX2. Anti-GAPDH was used as loading control. Fig. S2: SHOX2 cooperates with SOX5 and SOX9 to activate the Acan enhancer. Luciferase reporter activity of U2OS cells transfected with a reporter plasmid containing the Acan enhancer, renilla luciferase control plasmid and different combinations of SHOX, SHOX2 WT, SHOX2(p.L155V), SHOX2(p.Q234X), SOX5 and SOX9 expression plasmid as indicated. Fold-increase values were obtained by normalizing the relative luciferase units of each sample with the relative luciferase units of the sample transfected only with the reporter plasmid. All values represent the mean and standard deviation of three independent samples, with each sample assayed in triplicate. Significant p-values <0.001 obtained comparing different independent samples are indicated with two asterisks. Fig. S3: Expression of SHOX2 in different stages of the human fetal growth plate. Inmunohistochemistry performed with anti-SHOX2 antibody in normal fetal growth plates of 18, 27, 32 and 38-weeks. SHOX2 is expressed in the reserve (R), proliferative (P) and hypertrophic (H) chondrocytes. Images performed at 10× magnification. Fig. S4: SHOX2 is coexpressed with SHOX, SOX5, SOX6 and SOX9 in the 38-week human fetal growth plate. Immunohistochemistry per [file pone.0083104.s001.pdf]

Fig.S1

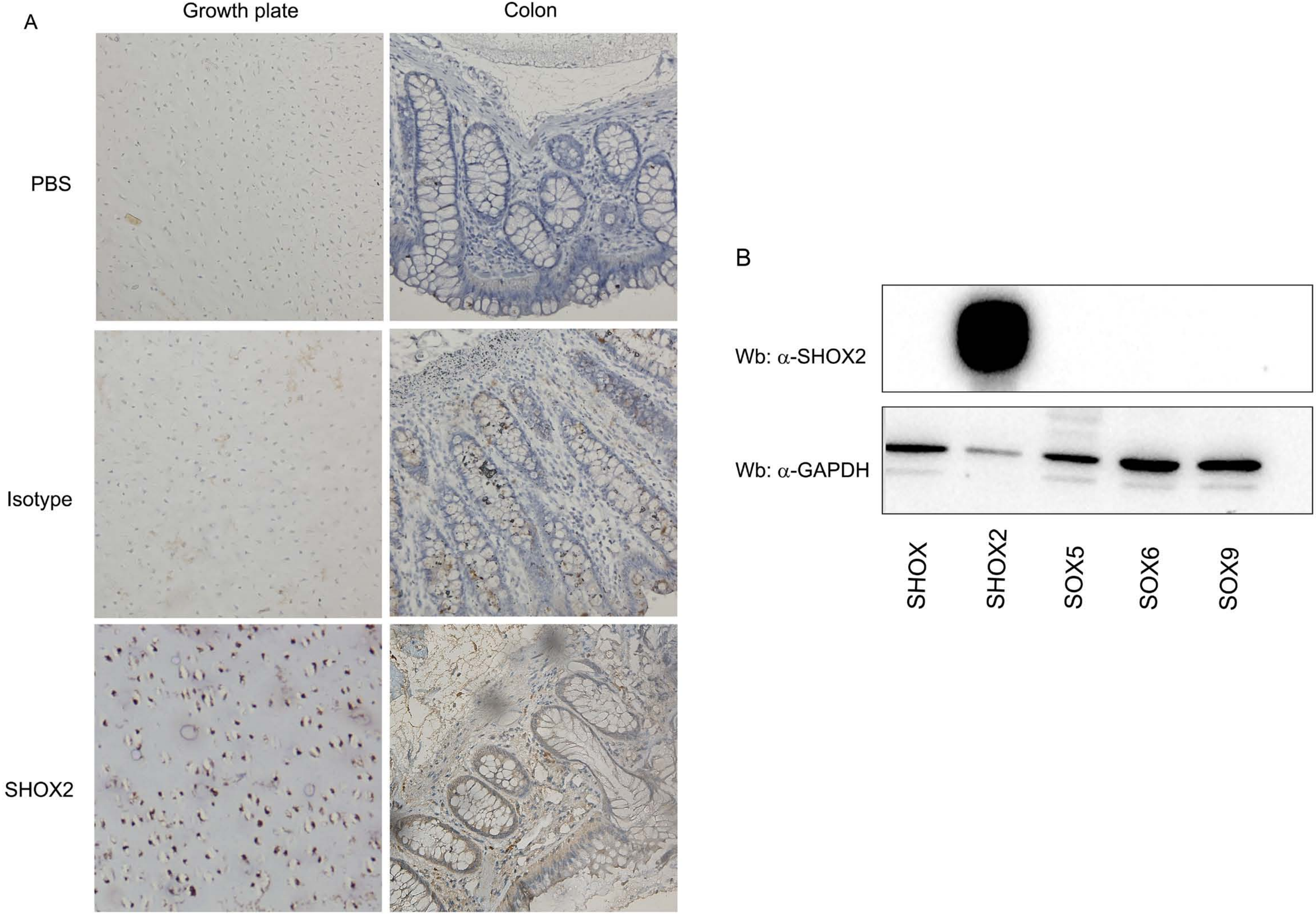
A
Growth plate
Colon
PBS
Isotype
SHOX2
B
Wb: α-SHOX2
Wb: α-GAPDH
SHOX
SHOX2
SOX5
SOX6
SOX9

Fig. S2

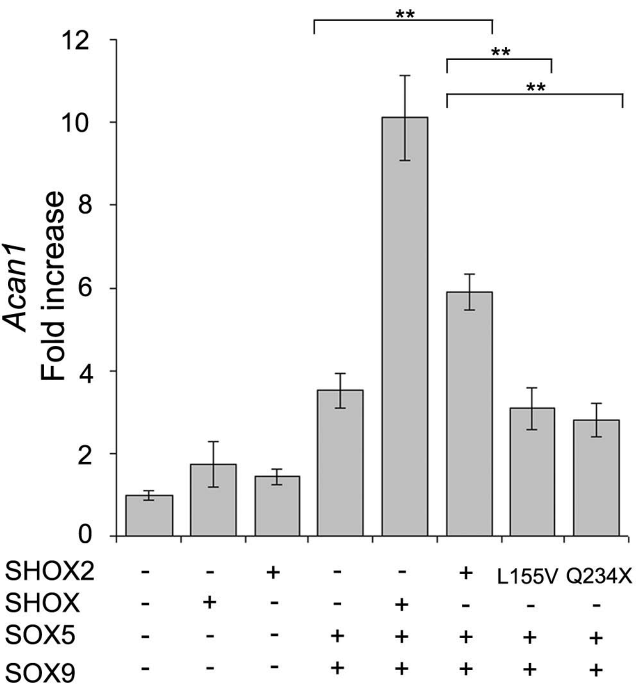
**
**
**
12
10
8
6
4
2
0
Acan1
Fold increase
SHOX2 - - + - - + L155V Q234X
SHOX - + - - + - - -
SOX5 - - - + + + + +
SOX9 - - - + + + + +

Fig S3

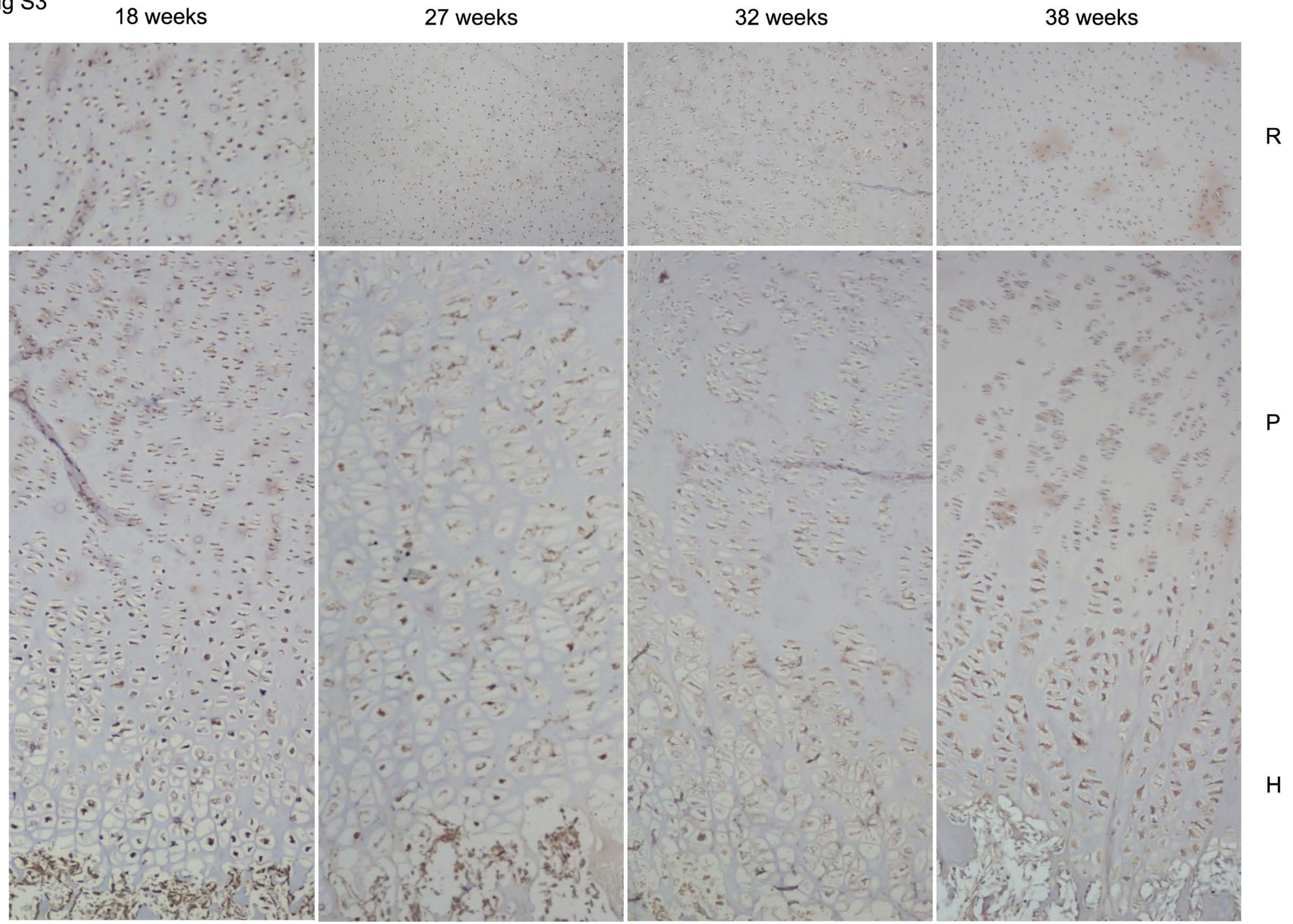

Fig.S4

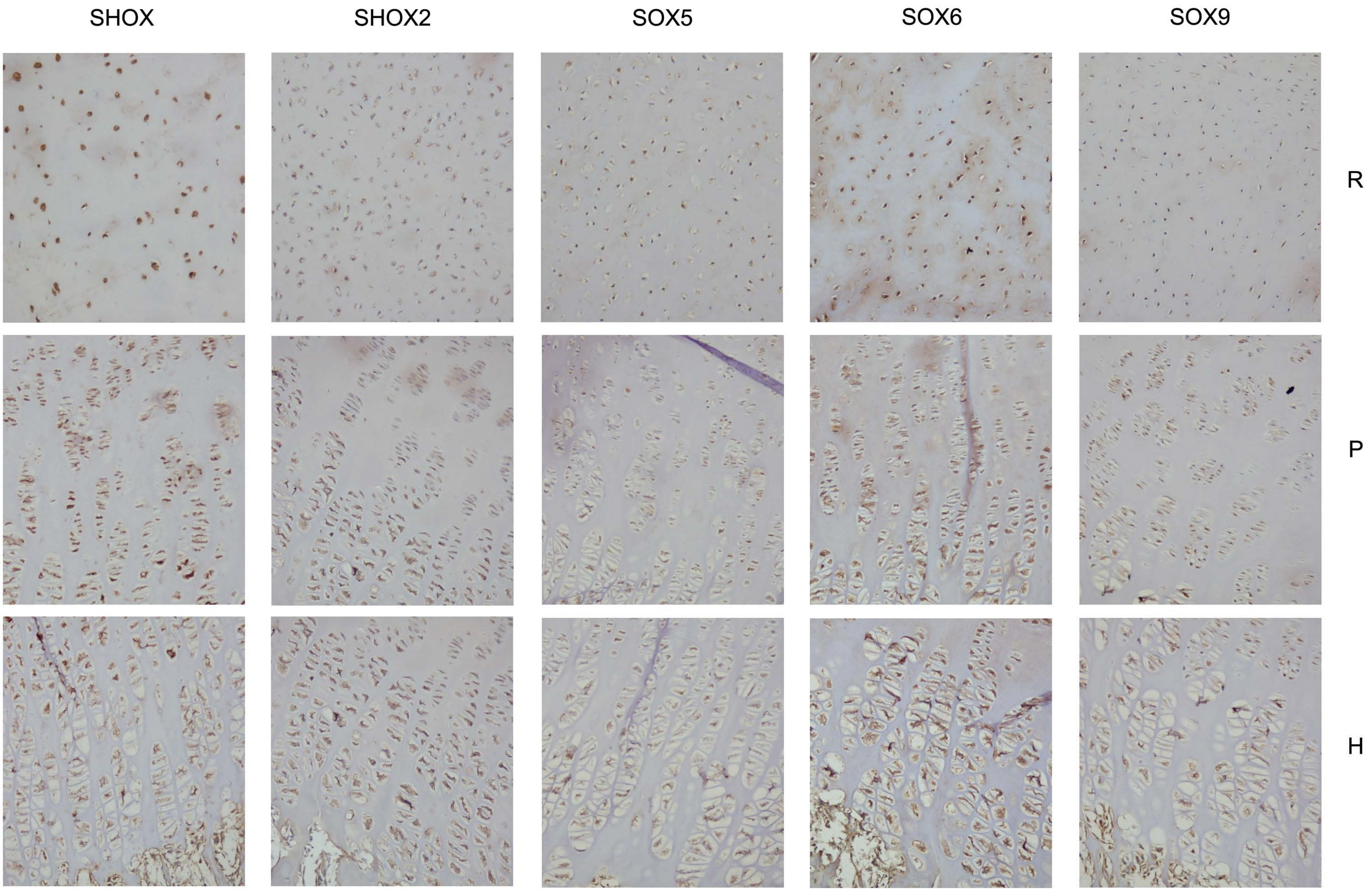

Table S1: Oligonucleotide sequences for the amplification of the SHOX2a cDNA and the cloning of the SHOX2 constructs. The incorporated enzymes sites are indicated in small letters. SHOX2a PCR A antisense oligonucleotide and SHOX2a PCR B sense oligonucleotide flank the sequence to be eliminated.

Constructs	Restriction enzyme sites	Sense oligonucleotide 5'-3'	Antisense oligonucleotide 5'-3'
SHOX2a PCR A	-	ATGGAAGAACTTACGGTTACGG	GCGCTGACTGCCTCCACAGGGGCCTCGACTTTC
SHOX2a PCR B	-	CTTTCAGCTCCGGGGACACCTCCGTCAGTCGCG	CTCACAGACCCAGGGCTGC
SHOX2a PCR ligation AB	-	ATGGAAGAACTTACGGTTACGG	CTCACAGACCCAGGGCTGC
pGBT9:SHOX2a	EcoRI/BamHI	GAgaattcATGGAGGACGAAGGC	ATggatccTCACAGACCCAGGGCTGC
pACT2:SHOX2a	BamHI/XhoI	ATggatccCGATGGAAGAACTTACGGTTACGG	ActcgagCTCACAGACCCAGGGCTGC
pGBT9:SHOX2b	EcoRI/BamHI	GAgaattcATGGAGGACGAAGGC	ATggatccTCACAGACCCAGGGCTGC
pACT2:SHOX2b	BamHI/XhoI	ATggatccGGATGGAGGACGAAGGCC	ActcgagCTCACAGACCCAGGGCTGC
pACT2:SHOX2(139-331)	BamHI/SalI*	GggatccGTAAGCAGAGGCGAAGTCGGAC	CgtcgacTCACAGACCCAGGGCTGCGGC
pACT2:SHOX2(200-331)	BamHI/SalI*	GggatccGTAATCAACTCCATAAAGGTG	CgtcgacTCACAGACCCAGGGCTGCGG
pcDNA3-HA:SHOX2	XbaI/EcoRI	ATtctagaGCGATGGAAGAACTTACG	TgaattcCTCACAGACCCAGGGCTGC
pcDNA3-FLAG:SHOX2			

* pACT2 vector was cut with BamHI/XhoI enzymes and ligated with the SHOX2 fragment cut with BamHI/SalI.

Table S2: Oligonucleotide sequences for the generation of various SHOX2 fragments and SHOX2 missense mutants. The mutated site is indicated by a small letter.

**Construct**	**Construct template**	**Mutation introduced**	**Sense oligonucleotide (5'- 3')**
pACT2:SHOX2(1-138)	pACT2:SHOX2	p.K139X	CCAGACCAAAATCtAGCAGAGGCGAAG
pACT2:SHOX2(1-200)	pACT2:SHOX2	p.Q201X	GAAAACAAGAAAATtgACTCCATAAAGGTGTTC
pACT2:SHOX2(139-200)	pACT2:SHOX2(139-331)		
pGBT9:SHOX2(K139E)	pGBT9:SHOX2	p.K139E	GGCCAGACCAAAATCgAGCAGAGGCGAAG
pACT2:SHOX2(K139E)	pACT2:SHOX2		
pGBT9:SHOX2(L155V)	pGBT9:SHOX2	p.L155V	GGAACAACTCAATGAGgTGGAGAGGCTTTTTGACG
pACT2:SHOX2(L155V)	pACT2:SHOX2		
pGBT9:SHOX2(A193P)	pGBT9:SHOX2	p.A193P	GGTTTCAAAATCGAAGAcCTAAATGTAGAAAAC
pACT2:SHOX2(A193P)	pACT2:SHOX2		
pGBT9:SHOX2(R196C)	pGBT9:SHOX2	p.R196C	CGAAGAGCTAAATGTtGcAAACAAGAAAATCAACTCC
pACT2:SHOX2(R196C)	pACT2:SHOX2		
pGBT9:SHOX2(R218Q)	pGBT9:SHOX2	p.R218Q	GCCAGTTTGAAGCTTGTcaAGTCGCACCTTATGTC
pACT2:SHOX2(R218Q)	pACT2:SHOX2		
pGBT9:SHOX2(A269D)	pGBT9:SHOX2	p.A269D	CCTGCATCCGCACCTGGaCGCGCACGCGCCCTAC
pACT2:SHOX2(A269D)	pACT2:SHOX2		
pGBT9:SHOX2(R319P)	pGBT9:SHOX2	p.R319P	CTCCAGCATCGCCGATCTCccACTGAAAGCCAAAAAG
pACT2:SHOX2(R319P)	pACT2:SHOX2		
pcDNA3-HA:SHOX2(L155V)	pcDNA3-HA:SHOX2	p.L155V	GGAACAACTCAATGAGgTGGAGAGGCTTTTTGACG
pcDNA3-HA:SHOX2(Q234X)	pcDNA3-HA:SHOX2	p.Q234X	GGATGCCATTTCAGtAGGATAGTCATTGCAACG

Table S3: Oligonucleotide sequences for the mutation screening of the coding exons and intron/exon boundaries of *SHOX2*.

***SHOX2* Exon**	**Oligonucleotide sequence**		**Ann. Temp. (ºC)**	**DMSO (5%)**	**Size (bp)**
	**Sense (5´- 3´)**	**Antisense (5´- 3´)**			
1	TGAGCGCCGGGCTGACGTGC	CCGGGGGTCAGTCAGGTCGT	66	+	396
2	GGATTTGCTGTGCTGTTTTCG	CTCTTTCCTTCTCATCTTACACC	57	-	426
3	GTTTAACTTTGGAATTTGAGGCTGT	CCAAACTTTAGGACTCCATTAACA	54	-	379
4	AGTCCATTTTCAAAGGAATTCG	GGGCTCAGAGACAGGTGATGTT	57	-	280
5	AAATGCCCTCTCCCCGTGCC	GATAGGGGACGAGGGATGGT	64	+	532

Table S4: Oligonucleotide sequences, PCR conditions and amplicon sizes of the *SHOX2* microsatellite markers. Microsatellites are listed in order from telomere to centromere.

Microsatellite marker	Repeat Unit	Oligonucleotide sequence		Size range	Ann. Temp.
		Sense (5'-3')	Antisense (5'-3')	(bp)	(ºC)
D3S3692	(CA)	CCATGCTCGTGGTAGAAGTC	ACCTAGCCTCAGAAGGCATC	190-204	55
D3S4638	(CA)	GATCCAGCAAACAAGACTAGG	CATATCTTTTGGACTCAAGGG	222-254	55
D3S4639	(AAAT)	CACAGAACTATTTTGAATTGG	CAGAATGCCTACTTTACTCAC	244-256	48
D3S4640	(TG)	GACTCAATTGGTTAGCTGTG	TGAATATCCTAATGCCTGTG	241-247	51

Table S5: *SHOX2* self-designed MLPA. Chromosomal and *SHOX2* location, probe lengths and ligation site sequences are indicated for the *SHOX2* and three control fragments.

Probe	Chromosome Location	*SHOX2* Location	Length (bp)	Sequence at Ligation Site
SHOX2- 1	3q25.32	Exon 1	93	GCGATGGAAGAACTTACGGCGTT-CGTCTCCAAGTCTTTTGACCAGAAAGTG
SHOX2- 2	3q25.32	Exon 2	117	CAAAATCAAGCAGAGGCGAAGTCGGACCAAT-TTCACCCTGGAACAACTCAATGAGCTG
SHOX2- 3	3q25.32	Intron 2	122	CAGAGCAGTCACGTTGAAGAGGAAA-AGTAATAATAGGCTGCCTTGGTCATCACAGC
SHOX2- 4	3q25.32	Exon 4	100	GAAGCTTGTAGAGTCGCACCTTATGTCA-ACGTAGGTGCTTTAAGGATGCCATTTCAGC
SHOX2- 5	3q25.32	Exon 5	133	GCTCTGTTTCTATTCTGTTGTCACCCTAGGA-TAGTCATTGCAACGTGACGCCCTTG
WBSCR1	7q11.23	-	89	GGTAGCTCTCGAGAATCTAGAGGTGG-ATGGGATTCCCGGGATGACTTC
RAI1	17p11.2	-	112	GGCAAGGAAAAGTGGCCTTTTGCGTGACA-CAGAAGTGGACACATCATTCTGATCTCAGGGCTCT
MAPT	17q21.31	-	128	CCTGGGACTTTAGGGCTAACCAGTTCTCTTTGTA-AGGACTTGTGCCTCTTGGGAGACGTCCACCCGTTTC
